# Quercetin suppresses ROS production and migration by specifically targeting Rac1 activation in gliomas

**DOI:** 10.3389/fphar.2024.1318797

**Published:** 2024-01-31

**Authors:** Rafia A. Baba, Hilal A. Mir, Taseem A. Mokhdomi, Hina F. Bhat, Ajaz Ahmad, Firdous A. Khanday

**Affiliations:** ^1^ Department of Biotechnology, University of Kashmir, Srinagar, India; ^2^ Cancer Diagnostic & Research Centre, Department of Immunology and Molecular Medicine, Sher-i-Kashmir Institute of Medical Sciences, Srinagar, India; ^3^ Departments of Ophthalmology, Columbia University, New York, NY, United States; ^4^ Department of Clinical Pharmacy, College of Pharmacy, King Saud University, Riyadh, Saudi Arabia

**Keywords:** quercetin, ROS, cell proliferation, cell migration, P66shc, oxidative stress

## Abstract

P66Shc and Rac1 proteins are responsible for tumor-associated inflammation, particularly in brain tumors characterized by elevated oxidative stress and increased reactive oxygen species (ROS) production. Quercetin, a natural polyphenolic flavonoid, is a well-known redox modulator with anticancer properties. It has the capacity to cross the blood–brain barrier and, thus, could be a possible drug against brain tumors. In this study, we explored the effect of quercetin on Rac1/p66Shc-mediated tumor cell inflammation, which is the principal pathway for the generation of ROS in brain cells. Glioma cells transfected with Rac1, p66Shc, or both were treated with varying concentrations of quercetin for different time points. Quercetin significantly reduced the viability and migration of cells in an ROS-dependent manner with the concomitant inhibition of Rac1/p66Shc expression and ROS production in naïve and Rac1/p66Shc-transfected cell lines, suggestive of preventing Rac1 activation. Through molecular docking simulations, we observed that quercetin showed the best binding compared to other known Rac1 inhibitors and specifically blocked the GTP-binding site in the A-loop of Rac1 to prevent GTP binding and, thus, Rac1 activation. We conclude that quercetin exerts its anticancer effects via the modulation of Rac1-p66Shc signaling by specifically inhibiting Rac1 activation, thus restraining the production of ROS and tumor growth.

## Introduction

Gliomas are the most common brain tumors, accounting for almost 30% of primary brain tumors and 80% of all malignant tumors. Gliomas have disturbed redox homeostasis, resulting in the activation of reactive oxygen species (ROS)-mediated cell survival and tumor growth signaling pathways (Olivier et al., 2021). P66Shc is an oxidative stress regulating protein that, along with P52Shc and P46Shc, belongs to the ShcA family of proteins. P66Shc plays a dual role in cell growth, regulating both cell proliferation and cell apoptosis by modulating ROS levels in cells (Mir et al., 2020). In response to oxidative stress conditions, p66Shc promotes apoptotic signals by undergoing ser36 phosphorylation at its CH2 domain (Mir et al., 2022). However, when stimulated by growth factors, p66Shc gets tyrosine phosphorylated and promotes mitogenic signals. P66Shc, in response to epidermal growth (EGF) stimulation, promotes cell proliferation, cell migration, and cell invasion by regulating the activity of small GTPases (Bhat et al., 2015). P66Shc overexpression has been correlated with the metastatic activity of various cancers. In addition, p66Shc, in association with SNTA1 and Grb2, has been reported to enhance the metastatic activity of breast cancers by enhancing Rac1 activity (Ali et al., 2021; Ali et al., 2022).

Rac1, a member of small monomeric GTP-binding proteins of the Rho (Ras homology) family, is a well-established regulator of ROS levels (Liang et al., 2021). To maintain the normal redox state of cells, the activation of Rac1 levels must be regulated precisely. Rac1 has two key functions: it regulates the organization of the actin cytoskeleton and controls the release of superoxides in cells via the modulation of the activity of the NADPH oxidase enzyme (Ma et al., 2023). A direct link between the activation of Rac1 and ROS production has been observed. Rac1 showed a direct correlation with ROS to promote cell migration of head and neck squamous cell carcinoma (Chen Y-H. et al., 2023). Apart from this, Rac1 has a role in diverse cellular processes, for example, membrane trafficking [5], hypoxia-stimulated breast cancer cell migration, cell adhesion, and cell proliferation (Chen M. et al., 2023; Bogucka-Janczi et al., 2023; Yang et al., 2023). Rac1 also has a role in cancer initiation, progression, invasion, and metastasis (Zhang et al., 2023). The overexpression of Rac-1 has been associated to the development of cancers such as testicular cancer, head and neck squamous cell cancer, colorectal, pancreatic, and breast cancer, and leukemia (Ramos et al., 2022; An et al., 2023; Gupta et al., 2023; Nakhaei-Rad et al., 2023).

Immense complexity of the human brain has made the treatment of gliomas an exigent task. Chemotherapeutics, though effective in treating cancer, show undesirable effects and are highly expensive. On the other hand, natural products have played a significant role in cancer drug discovery and have proven advantageous over conventional synthetic molecules. The chemopreventive properties of natural bioactive products have been increasingly important to researchers in the field of medicine in recent years (Harvey, 2008; Shen, 2015). It is believed that regular intake of vegetables and fruits can aid in preventing gliomas (Zhang et al., 2022). One possible reason is the presence of dietary antioxidants, for example, flavonoids in plant products since these are capable of decreasing oxidative stress and related damages (Mahomoodally et al., 2018; Slika et al., 2022; Hassan et al., 2023). The beneficial effects are proclaimed because of their capability to scavenge free radicals and reactive oxygen species. It is believed that free radicals and ROS induce DNA damage, cellular inflammation, and oxidative stress, ultimately leading to malignancies (Devika et al., 2022; Dewanjee et al., 2022). Therefore, it is likely that strengthening the antioxidant defense with an exogenous antioxidant might diminish these processes by decreasing the reactive species. Pronounced effects against these pathologies and cancer can, thus, be expected from treatment with antioxidants.

Quercetin is a dietary polyphenolic compound having high antioxidant activity and is chemically composed of three benzene rings and five hydroxyl groups. The sources of quercetin include apples, buckwheat, onions, and citrus fruits. Quercetin, as an antioxidant, has been suggested to have antidiabetic, anti-inflammatory, antioxidant, antimicrobial, anti-Alzheimer’s, antiarthritic, cardiovascular, and wound-healing effects (Alizadeh and Ebrahimzadeh, 2022; Michala and Pritsa, 2022). In addition, quercetin also shows antitumor properties against different cancers including pancreatic cancer, colon cancer breast cancer, and ovarian cancer (Salehi et al., 2020). The concentration at which quercetin exhibits anticancer activities under *in vitro* conditions ranges from 10 μM to 200 µM depending on the cancer cell type (Kedhari Sundaram et al., 2019; Salehi et al., 2020). The pharmacokinetic effects of intravenous quercetin injections were also studied in phase I clinical trials in 51 patients at doses of 60–2000 mg/m^2^. The study showed that a dose of 945 mg/m^2^ was safe, beyond which vomiting, high blood pressure, nephrotoxicity, and decreased serum potassium were reported (Ferry et al., 1996). Though quercetin has been shown to have anticancer activities, the molecular mechanisms behind are still inconclusive. Considering the facts that p66Shc regulates ROS-mediated tumor formation and quercetin being an antioxidant possessing anticancer properties, we were intrigued to explore its effects on p66Shc/Rac1-mediated ROS production and cell migration in an attempt to elucidate its possible mechanism of action.

## Materials and methods

### Chemicals, plasmids, antibodies, and reagents

Quercetin (3, 3′, 4′, 5, 7-pentahydroxyflavone) was purchased from Sigma (6151-25-3) and was dissolved in dimethyl sulfoxide (DMSO) before use. The final concentration of DMSO did not exceed 0.1% (v/v) throughout the experiments.

Xpress-tagged P66Shc WT cDNA in the PCDNA 3.1/His A and Rac1 vector were gifted by Dr. Shaida Andrabi.

Antibody to Shc (BD BioSciences, 610082), anti-vinculin (Upstate Biotechnology Inc., 05-386), anti-Rac1 (Millipore, 23A8), anti-β-actin (Sigma, A1978), and horseradish peroxidase-conjugated secondary antibodies (Sigma-Aldrich, OR03L) were used.

### Cell culture and treatment

C6 (CCL-107) rat glioma and IMR-32 (CCL-27) human neuroblastoma cells were procured from the National Centre for Cell Science (NCCS), India, and were grown up to a number of 50 passages. These cells were grown in Dulbecco’s Modified Eagle’s Medium (DMEM) (Sigma) supplemented with 10% fetal bovine serum (Sigma) and antibiotics (100 U/mL penicillin and 100 μg/mL streptomycin). All the cells were maintained at 37°C in a 5% CO_2_ incubator. C6 and IMR-32 cells were selected for this because of significant endogenous expression levels of the p66Shc protein. However, based on better transfection efficiency, most of the experiments were carried out in C6 cells.

Cells after attaining 70%–80% confluence were treated with various concentrations of quercetin (in DMSO) ranging from 25 μM to 200 µM for different intervals of time (0, 12, 24, 48, and 72 h). Quercetin concentrations were selected based on some previous studies which had reported its antitumor properties. To check for the involvement of ROS, N-acetylcysteine (NAC), which is a known ROS scavenger, was added to the cells either alone or in combination with quercetin. Vehicle controls were cells treated only with DMSO.

### Transfection

For transient transfection, cells were grown overnight in 10 cm dishes (Thermo Fisher Scientific (Carlsbad, CA, United States)) up to 70% confluence. The next day, complete media were removed, and serum-free media were added followed by transient transfection using the cationic lipid-based Lipofectamine 2000 (Invitrogen, United States) transfection reagent. Transfection was performed according to the protocol provided by the manufacturer; two microcentrifuge tubes with an equal volume of serum-free media were taken, and to one, Lipofectamine was added, while to another microcentrifuge tube, DNA along with P2000 was added. DNA mixture was then added dropwise to Lipofectamine tube, mixed, and incubated for 15–20 min at room temperature. The mixture was then dropwise added to cell culture dishes and incubated overnight. After transfection, the cells were analyzed for different time points (discussed in results) and were harvested or assayed as per the protocol.

### Cell viability

Cell viability was monitored using MTT (3-(4, 5-dimethylthiazol-2-yl)-2, 5-diphenyltetrazolium bromide) colorimetric assay. C6 and IMR-32 cells were seeded in 96-well plates (10^4^ cells per well) and cultured for different time intervals (12, 24, 48, and 72 h), followed by the treatment with varying concentrations of quercetin (25 μM, 50 μM, 100 μM, and 200 µM). Thereafter, the culture medium was aspirated, and 100 μL of MTT dye (1 mg/mL in PBS) was added into every well and incubated at 37°C for 3–4 h.

Acidified isopropanol (0.1N HCl) or DMSO was added to each well to solubilize the blue formazan crystals (if formed). Absorbance was read at 570 nm using a microplate reader (Epoc Biotech). The index of cell viability was determined by measuring the OD of the color produced by the reduction of MTT dye, compared with that of the untreated control cells [(OD of test compound-treated cells/OD of solvent-treated cells) ×100].

### Immunoblotting

C6 cells were seeded in 100 mm dishes to the density of 2.2 × 10^6^ per dish and transfected with the desired plasmid constructs. Transfected cells were treated with different doses of quercetin for specific time points depending on the experiments. Cells were then collected by trypsinization, washed with PBS, and lysed in lysis buffer [150 mM NaCl, 20 mM Tris-HCl (pH 7.4), 2 mM ethylenediaminetetraacetic acid (EDTA), 1% Nonidet P-40, 20% glycerol, 1 mM phenylmethylsulfonyl fluoride, and 5 mM NaF and containing a protease inhibitor cocktail]. The cell lysates (20 μg–40 μg) were solubilized in sample buffer [50 mM Tris-HCl (pH 6.8), 10% glycerol, 4% SDS, 5% β-mercaptoethanol, and 0.00125% bromophenol blue] and boiled for 5 min. Then, 30 μg of total protein from each cell lysate was resolved by and subjected to 10% sodium dodecyl sulfate-polyacrylamide gel electrophoresis (SDS-PAGE). Resolved proteins were then electro-transferred to the polyvinylidene difluoride (PVDF) membrane (Millipore). All the steps of Western blotting were followed as done previously [40]. Anti-Shc (1:3000), anti-Rac1 (1:1000), anti-vinculin (1:5000), and anti-β-actin (1:500) antibodies were used. Following transfer, immuno-detection was carried out, and the immunoreactive bands were visualized by chemiluminescence using the ECL system (Cell Biosciences).

### Rac1 activation assay

The amount of Rac1 activation (GTP-bound form) was determined using a commercial assay kit (Upstate Biotechnology, United States) that affinity precipitates GTP-Rac1 in cell lysates using the agarose conjugate-Rac1-binding domain of PAK. An activation assay was performed as per the protocol provided by the manufacturer, and the GTP-bound form of Rac1 associated with GST-PAK1 was detected using a monoclonal antibody against Rac1, also provided with the kit.

### Measurement of reactive oxygen species

Extracellular ROS generation in cells was assessed using the Amplex Ultra Red reagent (10 mM; Molecular Probes, Invitrogen). The Amplex Ultra Red reagent is a highly sensitive assay for detecting ROS levels. The Amplex Ultra Red reagent in the presence of peroxidase reacts with H_2_O_2_ in a 1:1 stoichiometry to produce the red-fluorescent oxidation product, resorufin. Cells seeded in six-well plates (0.3 × 10^6^ cells per well) were transfected with either p66Shc or Rac1 or both and then treated with quercetin. To each well, 50 μL of 10 mM Amplex UltraRed reagent and 100 μL of 10 U/mL horseradish peroxidase (HRP) were added. The plates were then kept in a CO_2_ incubator for 20 min incubation. The ROS generation was quantified by measuring fluorescence intensity (excitation, 568 nm; emission, 581 nm) using a spectrofluorophotometer (Shimadzu, RF-5301) against appropriate assay controls.

### Wound-healing assay

The effect of quercetin on the migratory potential of C6 cells was determined using wound-healing assay. In brief, the cells were seeded in a 24-well plate at a density of 1 × 10^5^ cells per well. At 70%–80% confluence, the cells were transfected with either p66Shc or Rac1 or both the plasmids together. A P-10 pipette tip was used to create a clean wound area across the center of the culture plate. The cells were then treated with 100 µM quercetin or 1 mM NAC; however, control cells were left untreated. The movement of the cells in the area of the wound was examined. The images of the distance migrated by the cells were taken under a light microscope (Olympus).

### Receptor structure extraction and ligand preparation

The X-ray-diffracted crystal structure of Rac1 protein was extracted from the Research Collaboratory for Structural Bioinformatics (RSCB) with accession number PDB ID 3TH5. It encompassed six helices and six small beta sheets and possessed a structure weight of 46561.09 A.U. (asymmetric unit), with a resolution of approximately 2.3 Å. The 3D ligand conformations were extracted from the PubChem database: (a) quercetin (Chem ID 5280343), (b) InSolution™ Rac1 Inhibitor (Chem ID: 16760632), (c) NSC 23766 (Chem ID: 16759159), (d) EHT 1864 (Chem ID: 9938202), (e) 6-mercaptopurine (Chem ID: 667490), and (f) ouabain (Chem ID: 439501).

### Molecular docking simulation

AutoDock 4.2 (Morris et al., 2009) was used to perform molecular docking simulations and to elucidate the binding conformations of the selected compound(s). Polar hydrogen atoms were added to the receptor protein and ligands; all bonds were allowed to rotate and were flexible during docking. Using AutoGrid, grid maps were generated by marking the grids around the whole receptor. Each grid was centered at the structure of the corresponding receptor. The grid dimensions were 30 Å * 30 Å * 30 Å with a spacing of 0.375 Å. The Lamarckian genetic algorithm was used for flexible ligand docking with 100 runs, a population size of 150, 2.5 * 106 evaluations, a maximum number of 27 * 103 iterations, an elitism value of 1, a mutation rate of 0.02, and a crossover rate of 0.80 (Mokhdomi et al., 2016). The binding conformations were then analyzed based on the energy values and the interacting residues. For comparing the molecular interaction of quercetin with Rac1 protein, the top five known Rac1 inhibitors were used: a) InSolution™ Rac1 Inhibitor (Chem ID: 16760632), b) NSC 23766 (Chem ID: 16759159), c) EHT 1864 (Chem ID: 9938202), d) 6-mercaptopurine (Chem ID: 667490), and e) ouabain (Chem ID: 439501). All molecular visualizations were carried out using Accelrys Discovery Studio Client 4.2.

### Molecular dynamics simulation

Using Desmond 2020.1 (Shaw et al., 2010), MD simulations were performed on the 3TH5 + quercetin complex. In this system, the OPLS-2005 force field (Bowers et al., 2006; Chow et al., 2008; Shivakumar et al., 2010) and explicit solvent model with the SPC water molecules (Jorgensen et al., 1983) were used in a period boundary salvation box with dimensions of 1.0 Å × 1.0 Å × 1.0 Å. In order to neutralize the electrical charge, sodium ions (Na^+^) were introduced. The metabolic environment was replicated by introducing a solution containing 0.15 M NaCl into the apparatus. Initially, the system underwent retraining by subjecting the protein–ligand complexes to an NVT ensemble for a duration of 10 nanoseconds. Following the preceding procedure, an NPT ensemble was used to conduct a brief 12-ns run of equilibration and minimization. The NPT ensemble was established using the Nose–Hoover chain coupling method (Martyna et al., 1994). In each of the models, the temperature was varied while maintaining a constant relaxation time of 1.0 picoseconds and a pressure of 1 bar. The temporal increment used in the simulation was 2 femtoseconds. The Martyna–Tuckerman–Klein chain coupling scheme barostat technique (Martyna et al., 1992) was used to regulate the pressure. The period of relaxation was chosen as 2 picoseconds. In the computation of long-range electrostatic interactions, we used the particle mesh Ewald approach. The radius for the coulomb interactions was maintained at a constant value of 9 Å. The bonded forces for each trajectory were calculated using the RESPA integrator with a time step of 2 fs. The most recent manufacturing run was conducted with a temporal duration of one hundred nanoseconds per unit. To assess the stability of the molecular dynamics (MD) simulations, several key parameters were quantified, including the root mean square deviation (RMSD), radius of gyration (Rg), root mean square fluctuation (RMSF), and number of hydrogen bonds (H-bonds). The aforementioned values were used for the purpose of assessing the stability of the MD simulations.

### Statistical analysis

Statistical analyses were carried out using GraphPad Prism 6 (v 6.01). For dose- and time-dependent studies, a statistical analysis was carried out using one-way analysis of variance (ANOVA), with multiple comparisons using Dunnett’s *post hoc* test, and the level of significance was tested at a *p*-value ranging from 0.05 to 0.001. In comparative analysis, Student’s t-test (one tailed) was used. The level of significance was tested at 95% CI, *p* < 0.05. All the data are expressed as the mean ± SD of at least three independent experiments. Asterisks represent statistical significances and are indicated as follows: *p < 0.05, **p < 0.01, and ***p < 0.001.

## Results

### Quercetin inhibits the growth of C6 and IMR-32 cells in a dose-dependent but not time-dependent manner

**FIGURE 1:**
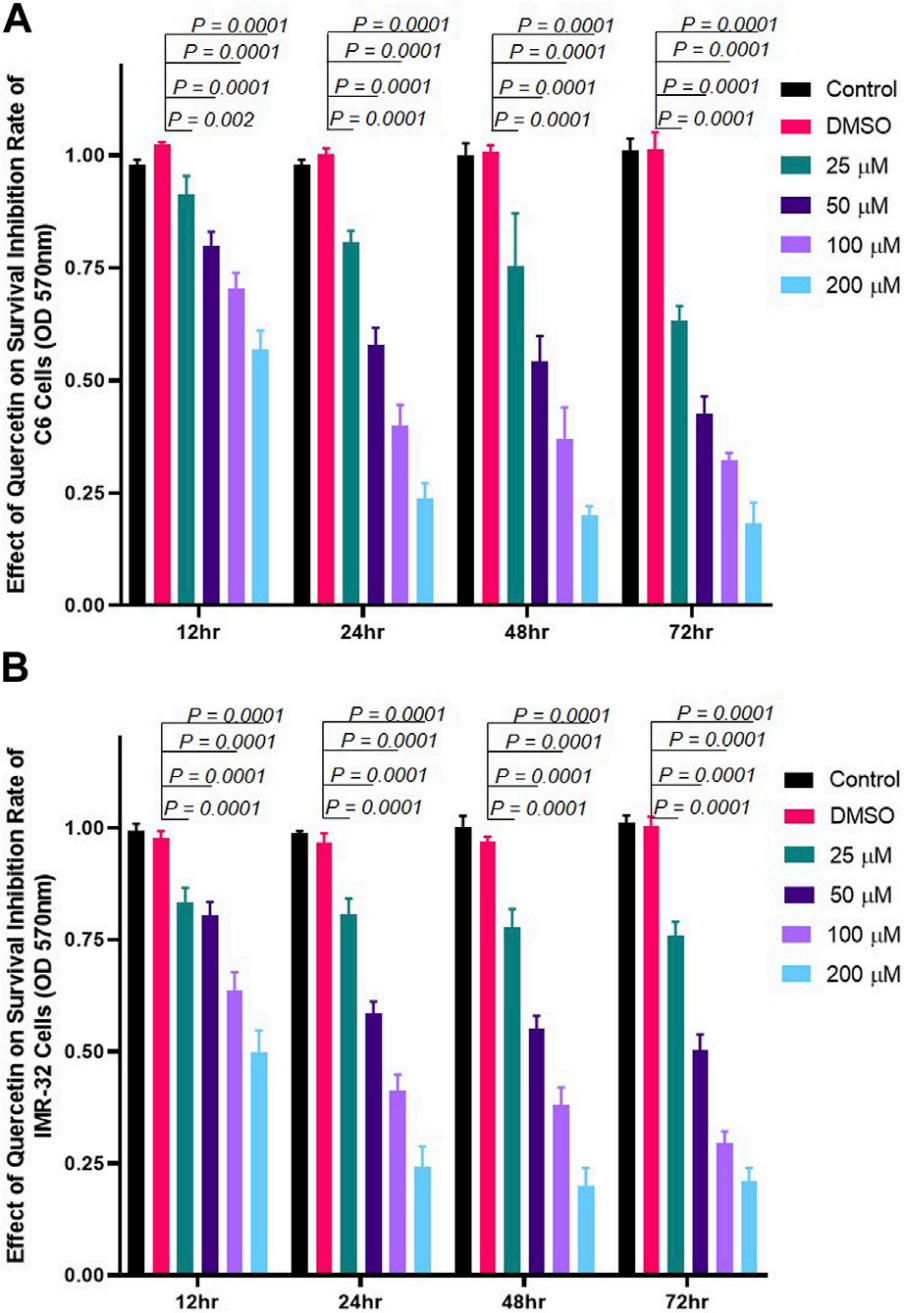
Quercetin treatment inhibits the proliferation of neuronal cells. Cells were incubated with different concentrations of quercetin (25 μM, 50 μM, 100 μM, and 200 µM) for 12, 24, 48, or 72 h, respectively. Control comprised cells without quercetin treatment. **(A)** Bar graph showing the effect of quercetin on rat glioma C6 cells in a concentration- and time-dependent manner. **(B)** Bar graph showing the effect of quercetin on human neuroblastoma IMR-32 cells in a concentration- and time-dependent manner. The data shown represent the mean ± standard deviation of three biological replicates. The statistical analysis was carried out using one-way ANOVA with multiple comparisons using Dunnett’s *post hoc* test to determine the level of significance. **p < 0.01; *** *p* < 0.0001.

The effect of quercetin on the viability of C6 and IMR-32 cells was evaluated using MTT assay. Cells were treated with increasing concentrations of quercetin (25 μM, 50 μM, 100 μM, and 200 µM) for different time points (12, 24, 48, or 72 h). We found that quercetin significantly inhibited the growth and proliferation of both C6 and IMR-32 cells in a dose-dependent manner but showed time dependence till 24 h only (doubling time of cells). The inhibition of proliferation beyond 24 h drug treatment showed little or no difference at each tested dose (Figure 1).

### Quercetin inhibits the endogenous and exogenous expression of p66Shc and Rac1 proteins

**FIGURE 2:**
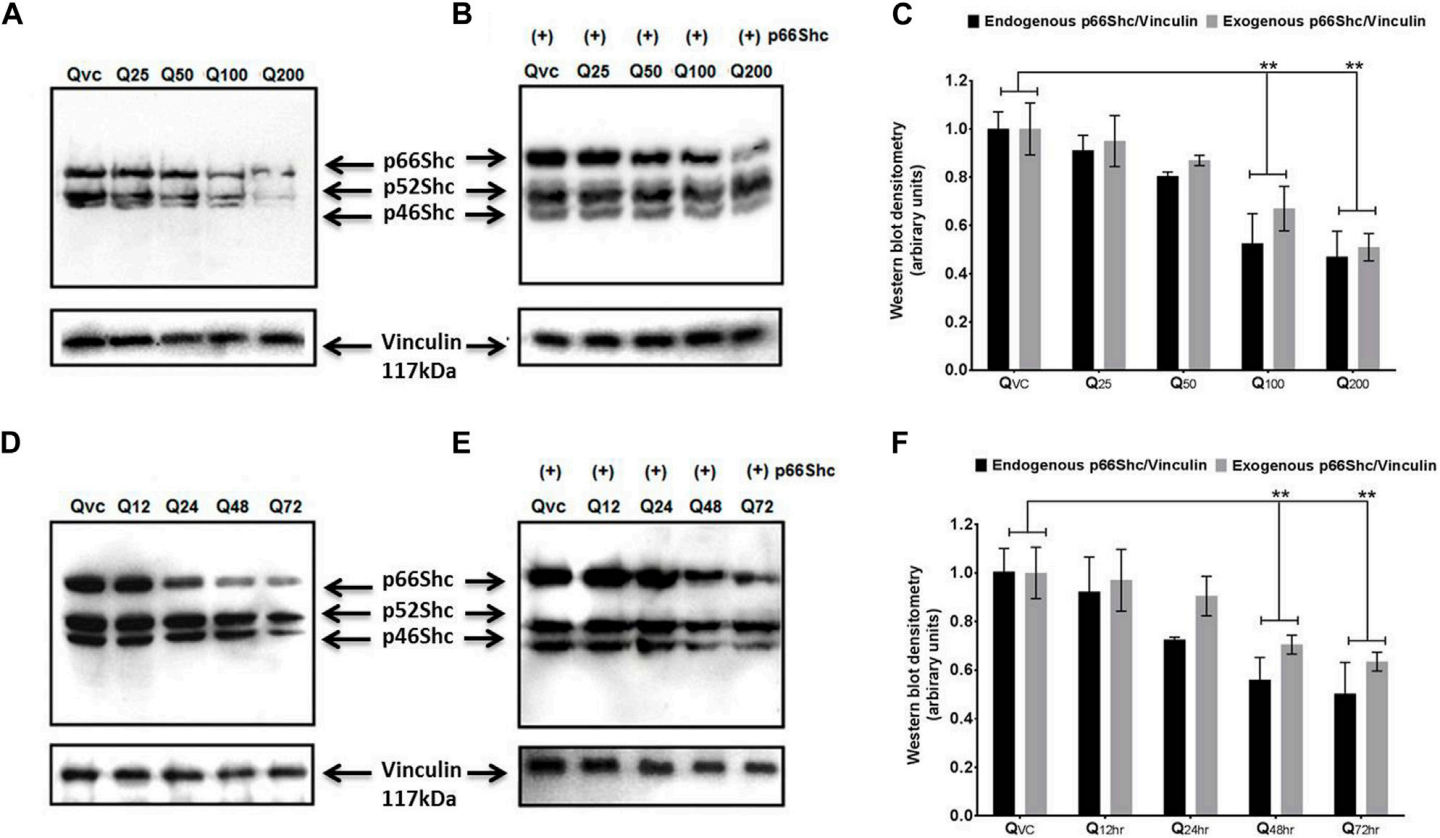
Quercetin decreases the expression levels of p66Shc. **(A)** Representative immunoblot showing the dose-dependent effect of quercetin for 24 h on endogenous p66Shc. **(B)** Immunoblot showing the effect of quercetin for 24 h on exogenous (+) p66Shc (externally transfected) in C6 cells. **(C)** Bar diagram (densitometric analysis) showing the fold change in the expression levels of endogenous and exogenous p66Shc in response to quercetin treatment. **(D)** Immunoblot showing the time-dependent effect of quercetin on the endogenous expression of p66Shc. **(E)** Immunoblot showing the time-dependent effect of quercetin on exogenous (+) p66Shc expression in cells treated with 100 µM quercetin. Also detected in the blot are two other Shc isoforms, i.e., p52Shc and p46Shc. QVC: vehicle control. Q25–Q200: 25 µM–200 µM concentrations of quercetin used. Q12 hr–Q 72 h: 100 µM concentration of quercetin used for 12 h–72 h. Lower panels represent the expression of vinculin protein used as loading control, in respective treatment groups. **(F)** Bar diagram densitometric analysis showing the fold change in the expression of p66shc in response to quercetin in a time-dependent manner. The results shown were repeated at least three times and are expressed as mean ± SD fold change over the control level set to 1 unit and normalized to total protein. The statistical analysis was carried out using one-way ANOVA with multiple comparisons using Dunnett’s *post hoc* test. **p < 0.01 represents significant variation compared to respective vehicle controls.

**FIGURE 3:**
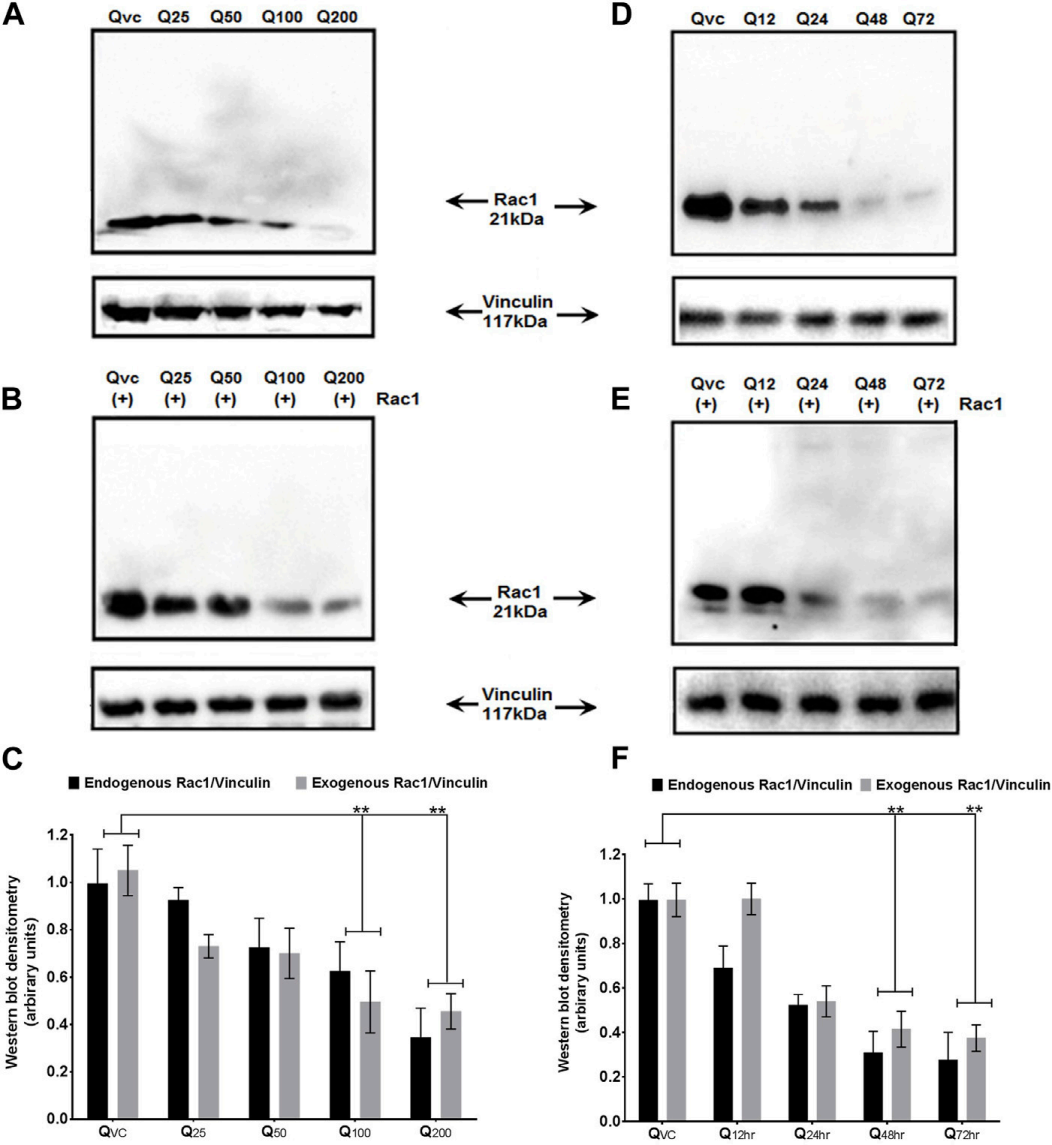
Effect of quercetin on the expression of Rac1 protein. **(A)** Western blot immunoblot showing a dose-dependent effect of quercetin on endogenous Rac1 at different time points in C6 glioma cells. **(B)** Western blot immunoblot showing a dose-dependent effect of quercetin on exogenous Rac1 at different time points in C6 glioma cells. **(C)** Bar diagram (densitometric analysis) showing the fold change in the expression levels of endogenous and exogenous Rac1 in response to quercetin treatment in a concentration-dependent manner. **(D)** Immunoblots showing the time-dependent effect of quercetin on the expression levels of endogenous Rac1 in C6 glioma cells and harvested after 24 h. **(E)** Immunoblots showing the time-dependent effect of quercetin on the expression levels of exogenous Rac1 in C6 glioma cells harvested after 24 h. QVC: vehicle control. Q25–Q200: 25 µM–200 µM concentrations of quercetin used. Q12 hr–Q72 h: 100 µM concentration of quercetin used for 12 hr–72 h. Lower panels represent the expression of vinculin protein used as loading control, in respective treatment groups. **(F)** Bar diagram (densitometric analysis) showing the fold change in the expression levels of endogenous and exogenous Rac1 in response to quercetin treatment in a time-dependent manner. The results shown were repeated at least three times and are expressed as mean ± SD fold change over the control level set to 1 unit and normalized to total protein. The statistical analysis was carried out using one-way ANOVA with multiple comparisons using Dunnett’s *post hoc* test. **p < 0.01 represents significant variation compared to respective vehicle controls.

Both p66Shc and Rac1 share a common activation pattern as both are being induced by cellular stress and show interdependence. The former acts as scaffolding protein to bring about the expression of Rac1, which, in turn, releases stress factors and induces the expression of p66Shc. To elucidate the effect of quercetin on p66Shc and Rac1 expression, C6 cells were transfected with p66Shc and Rac1, incubated with 25 μM, 50 μM, 100 μM, and 200 µM concentrations of quercetin, and harvested after 24 h incubation. After harvesting, cell lysates were titrated against anti-p66Shc and anti-Rac1 antibodies. The results indicate that quercetin inhibited the expression of endogenously and exogenously transected p66Shc/Rac1 expression in a dose-dependent manner (Figures 2A, B), (Figures 3A, B).

We also looked for the time dependence of quercetin treatment on the expression of p66Shc and Rac1 proteins. A consistent decrease in Rac1 expression was observed in both transfected and untransfected cells upon quercetin (100 µM) treatment (Figures 2D, E; Figure 3D; Figure 2E); however, the decrease in the expression of p66Shc was less prominent after 24 h.

### Quercetin downregulates p66Shc-Rac1-mediated production of reactive oxygen species

**FIGURE 4:**
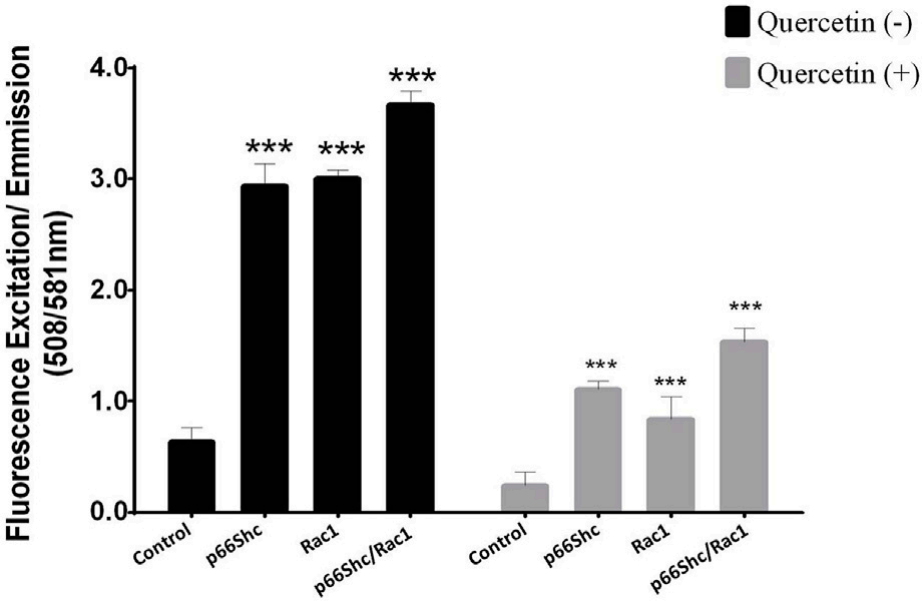
Quercetin decreases p66Shc/Rac1-mediated ROS production: comparison of ROS production in cells transfected either with p66Shc, Rac1, and/or both the plasmid constructs together following quercetin (100 µM) treatment. Controls represent untransfected cells. The results shown were repeated at least three times and are expressed as mean ± SD. The statistical analysis was carried out using one-way ANOVA with multiple comparisons using Dunnett’s *post hoc* test. *p < 0.05, **p < 0.01, and ***p < 0.00 represent significant variations compared to respective vehicle controls.

Both p66Shc and Rac1 are known to be involved in ROS generation and the regulation of cellular oxidative stress; hence, the effect of quercetin on ROS production via the p66Shc–Rac1 pathway was studied. Cells were transiently transfected with (i) p66Shc, (ii) Rac1, and/or (iii) p66Shc and Rac1 together. The Amplex UltraRed reagent was used to monitor extracellular ROS generation in C6 glioma cells, following quercetin treatment. The overexpression of p66Shc and/or Rac1 in cells resulted in a significant increase in ROS generation, which showed a peak when both the vectors were expressed together. However, the treatment of cells with quercetin not only reduced the levels of ROS (Figure 4) but also showed a significant inhibition of p66Shc/Rac1, irrespective of whether both the vectors are co-expressed or not.

### Quercetin prevents Rac1 activation in a dose-dependent manner

**FIGURE 5:**
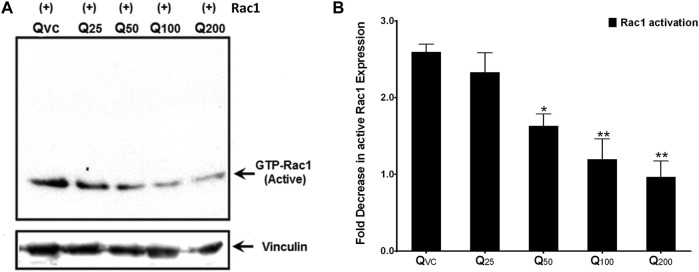
Quercetin decreases active Rac1 levels in c6 cells. **(A)** Representative immunoblot showing a decrease in active Rac1 levels upon quercetin treatment in immuno-precipitated lysates from C6 cells transiently transfected with the Rac1 plasmid construct. **(B)** Western densitometry quantifications showing fold decrease in active Rac1 upon quercetin treatment compared to control (untreated). The results shown were repeated at least three times and are expressed as mean ± SD. The statistical analysis was carried out using one-way ANOVA with multiple comparisons using Dunnett’s *post hoc* test. *p < 0.05, **p < 0.01, and ***p < 0.00 represent significant variations compared to respective vehicle controls.

Since a consistent decline in the expression of both p66Shc and Rac1 proteins as well as ROS production was observed upon quercetin treatment, we looked for the effect of quercetin on the activation of Rac1 to validate the specificity of quercetin action. C6 cells were transiently transfected with the Rac1 plasmid and treated with varying concentrations of quercetin. The pull-down experiments showed a 2–2.5-fold decrease in active Rac1 upon quercetin treatment (Figure 5) compared to untreated cells.

### Quercetin inhibits cell migration by limiting p66Shc-Rac1-mediated ROS production

**FIGURE 6:**
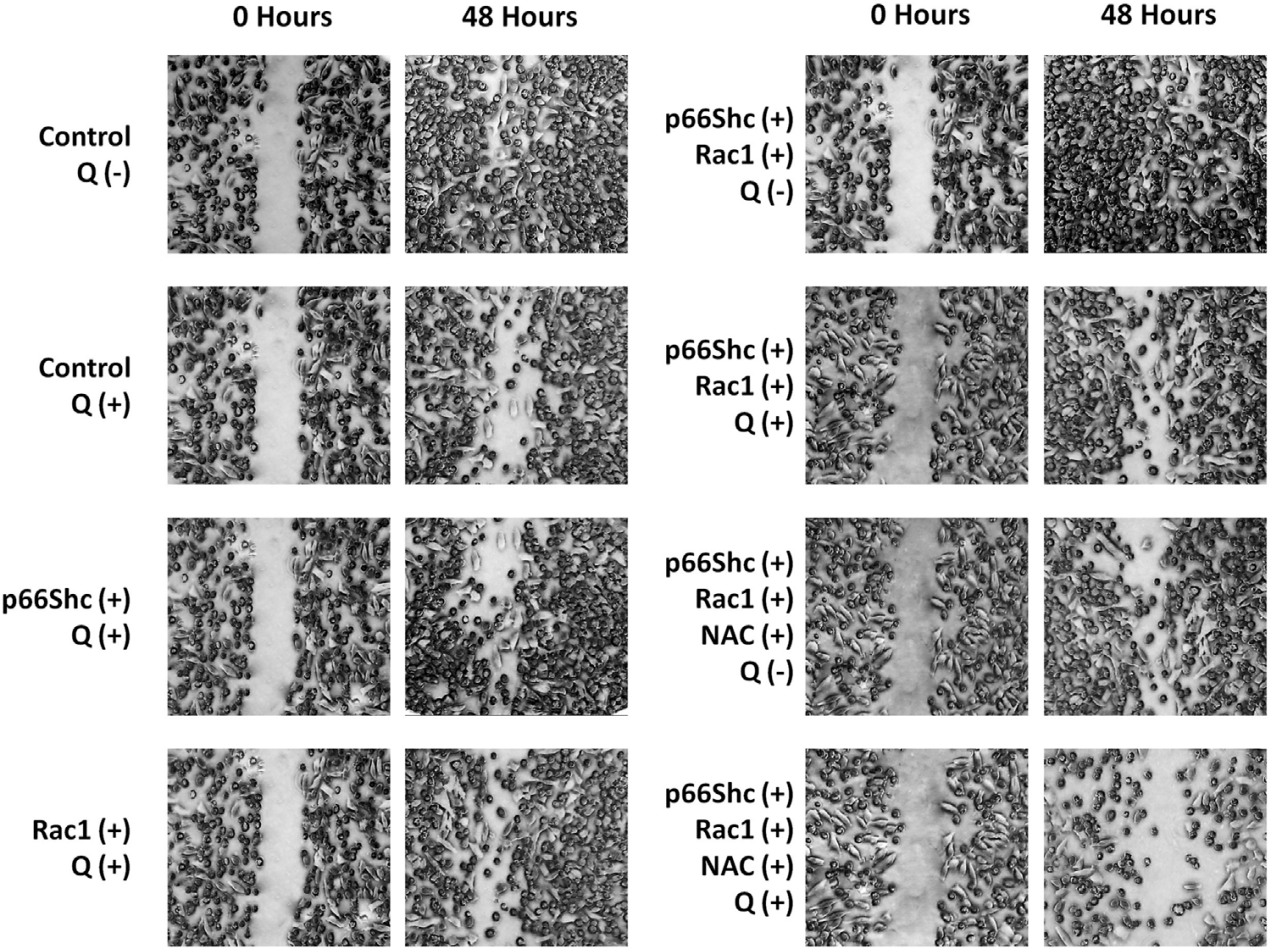
Quercetin inhibits cell migration in ROS-dependent manner. Representative photomicrographs depicting the effect of quercetin (100 µM) in the absence and presence of NAC (1 mM) on cell migration in cultures of C6 cells transfected with p66Shc and/or Rac1 plasmid constructs following scratch wound. Representative photographs illustrate the results of three independent experiments.

Cellular stress induced by ROS has been implicated in stimulating migration in benign tumor cells (Mir et al., 2020). Since ROS generation is primarily regulated by p66Shc-Rac1 signaling, we looked for the effect of quercetin on the migratory potential of gliomas using wound-healing assay by modulating p66Shc, Rac1, or ROS titer. Quercetin effectively decreased the migratory potential of cells, even after transfection with p66Shc or Rac1 (Figure 6), which otherwise increases cell proliferation (data not shown). When both of these proteins, viz., p66Shc and Rac1, were co-expressed in cells, wound healing conspicuously increased after 48 h following the wound. However, there was a noticeable decrease in cell migration in these cells after quercetin treatment. The effect of quercetin on cell migration was comparable to that of NAC (1 mM), a known ROS scavenger which showed a considerable decrease in wound healing at 48 h, thus pointing toward an ROS-dependent inhibition of cell migration by quercetin. The results were further corroborated by combinatorial treatment of quercetin and NAC on wound healing which drastically reduced cell migration across the wound area (Figure 6).

### Quercetin binds to the P-loop harboring the GTP-binding site to prevent Rac1 activation

**FIGURE 7:**
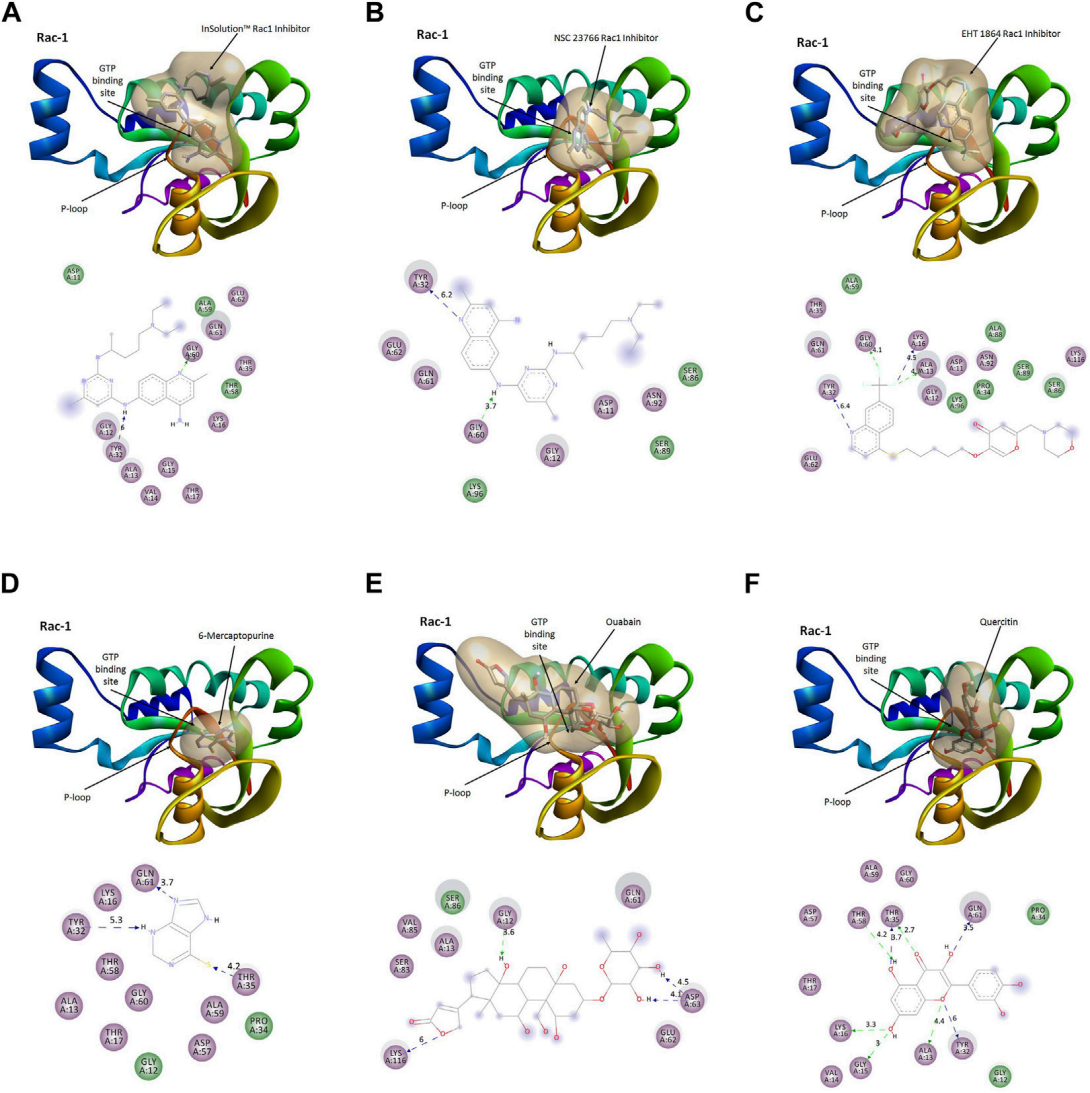
Comparative binding modes of Rac1 inhibitors and quercetin. The results of the molecular docking simulation of Rac1 protein with **(A)** InSolution™ Rac1 Inhibitor, **(B)** NSC 23766, **(C)** EHT 1864, **(D)** 6-mercaptopurine, **(E)** ouabain, and **(F)** quercetin showing respective binding modes of inhibitors targeting the GTP-binding site. Ligand interactions at the GTP-binding site of Rac1 are depicted in molecular models.

The activation of Rac1 is dependent on the binding of GTP to its nucleotide-binding pocket, stimulating effector proteins, and regulates cellular processes, viz., cell adhesion, cell proliferation, and ROS production. To evaluate the quercetin-mediated inhibition of Rac1 activation, we performed molecular docking simulation (MDS) of Rac1 with quercetin and the top five Rac1 inhibitors targeting the GTP-binding site in the P-loop of Rac1 (Figure 7). Quercetin showed the best binding (∆G = −9.86 kcal/mol) with the P-loop and specifically blocked the GTP-binding site (Ki_Quer_ = 58.82 uM) compared to other Rac1 inhibitors, viz., a) InSolution™ Rac1 Inhibitor (∆G = −4.88, Ki_InSol_ = 264.46 uM), b) NSC 23766 (∆G = −5.46; Ki_NSC_ = 99.15 uM), c) EHT 1864 (∆G = −5.22; Ki_EHT_ = 149.5 uM), d) 6-mercaptopurine (∆G = −5.66; Ki_Mer_ = 71.38 Um), and e) ouabain (∆G = −4.51; Ki_Ouab_ = 490.82 uM). The binding interactions of quercetin with the GTP-binding site of Rac1 comprised amino acids GLY12, ALA13, VAL14, GLY15, LYS16, and THR17 in the P-loop and ASP57, THR58, ALA59, GLY60, and GLN61 in the A-loop of Rac1. Quercetin showed the highest H-bonding (*n* = 5) compared to other Rac1 inhibitors with a bond energy of −11.65 kcal/mol. As docking studies showed significant binding affinity of Rac1 with quercetin, we further utilized molecular dynamics simulation to further evaluate the stability of the Rac1–quercetin complex.

### Quercetin targets Rac1 with high affinity and stability

**FIGURE 8:**
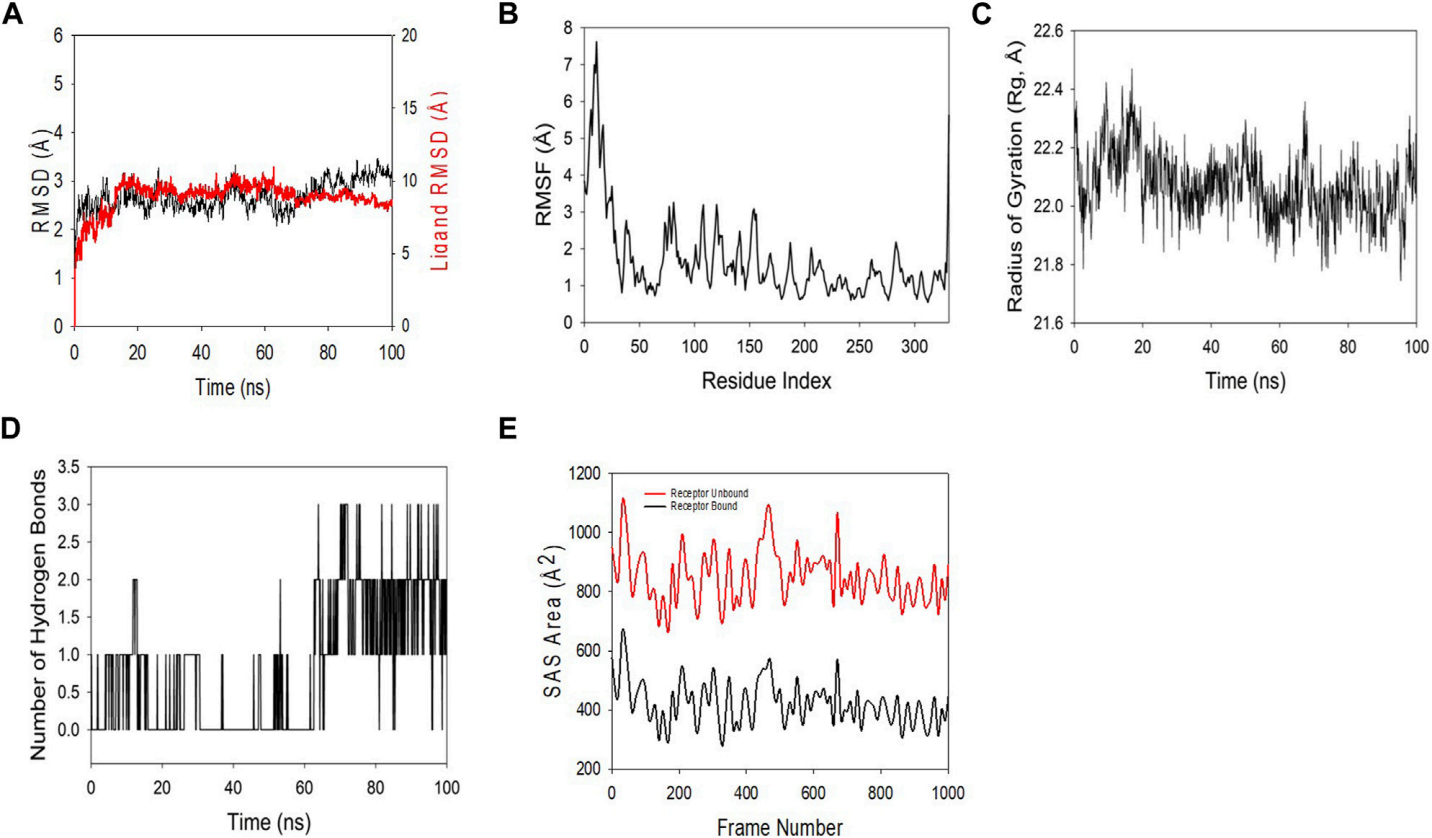
MD simulation analysis of the 100 ns trajectories of **(A)** the Cα backbone RMSD of 3TH5 bound to ligand quercetin. **(B)** RMSF of the Cα backbone of 3TH5 bound to ligand quercetin. **(C)** Cα backbone radius of gyration (Rg) of 3TH5 bound to ligand quercetin. **(D)** Formation of hydrogen bonds in 3TH5 bound to ligand quercetin. **(E)** Solvent-accessible surface area of 3TH5 bound to ligand quercetin.

MD and simulation studies were carried out in order to determine the stability and convergence of Rac1 + quercetin. Simulation of 100 ns displayed almost stable conformation while comparing the RMSD. The RMSD of the Cα-backbone of Rac1 + quercetin up to 60 ns exhibited dangling deviations, which later became more stable up to 100 ns (Figure 8A). A stable RMSD plot during simulation signifies a good convergence and stable conformations. Thus, it can be suggested that Rac1 was quite stable in complex with quercetin due to higher affinity. Moreover, RMSF plots displayed large spikes of fluctuation in Rac1 observed at the 1st to 40th residues, which may be due to the higher flexibility of the residues (Figure 8B), but no significant fluctuations were observed later on. Most of the residues less fluctuating during the entire 100 ns simulation (Figure S1) indicate the rigid amino acid conformations during the simulation time. Therefore, from RMSF plots, it can be suggested that the protein structure is rigid during simulation in ligand-bound conformations. Rg is the measure of compactness of the protein. In this study, the Rac1 + quercetin Cα-backbone displayed the lowering of Rg from 22.3 to 22.2 Å (Figure 8C). The lowering of Rg indicates the highly compact orientation of the protein in the ligand-bound state. The number of hydrogen bonds between the protein and the ligand suggests the significant interaction and stability of the complex. The number of hydrogen bonds between Rac1 and quercetin showed initially up to 65 ns one hydrogen bond observed on average, while later on, an addition of hydrogen bonds was observed up to 100 ns (Figure 8D). Followed by Rg analysis, patterns were also observed in the solvent-accessible surface area (SASA) in both ligand-bound and -unbound states. It is clearly visible from Figure 8E that in the unbound state, Rac1 displayed high surface area accessible to solvent in all the cases. The SASA value lowered as compared to the unbound state while bound with quercetin (Figure 8E). The overall study of Rg signifies that the ligand binding compels the respective proteins to become more compact.

Utilizing the MD simulation trajectory, the binding free energy along with other contributing energies in the form of MM-GBSA was determined for complex Rac1 + quercetin. The results (Table 1) suggested that the maximum contribution to ΔG_bind_ in the stability of the simulated complexes was due to ΔG_bind_Coulomb, ΔG_bind_vdW, and ΔG_bind_Lipo, while, ΔG_bind_Covalent and ΔG_bind_SolvGB contributed to the instability of the corresponding complexes. Furthermore, the Rac1 + quercetin complex has significantly higher binding free energies (Table 1). These results supported the potential of Rac1 + quercetin having high affinity of binding to the protein as well as efficiency in binding to the selected protein and the ability to form stable protein–ligand complexes.

**TABLE 1:** Binding free energy components for the 3TH5 + quercetin calculated from MM-GBSA.

Energy (kcal/mol)	3TH5 + quercetin
ΔG_bind_	−76.04 ± 2.63
ΔG_bind_Lipo	−23.96 ± 1.03
ΔG_bind_vdW	−51.10 ± 2.0
ΔG_bind_Coulomb	−8.12 ± 1.99
ΔG_bind_H_bond_	−0.41 ± 0.22
ΔG_bind_SolvGB	16.5 ± 1.09
ΔG_bind_Covalent	1.56 ± 1.2

## Discussion

RhoGTPase Rac1 protein, primarily involved in cell adhesion and motility in migrating cells, has been implicated in oxidative stress, enhancing the production of ROS and, in particular, ROS-mediated tumor inflammation (Azarova et al., 2022; Marwaha et al., 2022; Liao et al., 2023). Although the human body has an innate antioxidant defense mechanism to counter oxidative species in cells, it may not function once ROS exceeds a threshold level leading to cellular stress. The overproduction of ROS, particularly peroxides, tends to alter cell cycle phases, eventually leading to cancer (Mehraj et al., 2022a; Mehraj et al., 2022b; Chen J-S. et al., 2023; Liu et al., 2023). The situation becomes more vulnerable in metabolically active tissues such as the brain which, apart from being less equipped with innate antioxidant defense, also tend to resist the delivery of drugs due to the blood–brain barrier (Rinaldi et al., 2016). Augmenting the innate antioxidant defense with external antioxidants having the ability to cross the blood–brain barrier could, thus, be beneficial in the treatment of brain tumors (Mittal et al., 2023).

Quercetin is a powerful antioxidant and effective scavenger for highly reactive species such as hydroxyl radicals, peroxinitrite, and superoxide radicals and has the ability to cross the blood–brain barrier and, therefore, is a potential drug for brain tumors (Gibellini et al., 2011; Zhang et al., 2011; Asgharian et al., 2022). The effect of antioxidants such as quercetin on the regulation of the p66shc-Rac1 pathway which principally drives the generation of ROS apart from regulating apoptosis and/or cell proliferation has not been evaluated so far. Therefore, in this study, we explored the effect of quercetin on Rac1 signaling and tumor cell proliferation.

We show that quercetin effectively inhibited the proliferation of both human neuroblastoma cells (IMR-32 cells) and rat glioma C6 cells (Figure 1). The decline in cell proliferation was consistent with inhibition in the expression of p66Shc protein (Figure 2), pointing toward the modulation of RhoGTPase Rac1 signaling by quercetin. Quercetin not only inhibited the expression of intracellular p66Shc but also prevented the expression of the transfected p66Shc gene. P66Shc has been recognized as a ROS producer since it acts as an oxidoreductase enzyme capable of oxidizing cytochrome C and H_2_O_2_ production, resulting in the opening of permeability transition pores (PTPs) and release of cytochrome C (Giorgio et al., 2005). In cytosol, p66Shc acts as a switch in separating Sos1 from the Grb2/Sos1 complex to the Eps8/E3b1 complex, resulting in the activation of Rac1 and ROS production (Baba et al., 2012). The GTPase Rac1, once activated, triggers the production of ROS via a varied number of intracellular processes. The prime target of activated Rac1 is the plasma membrane-NADPH oxidase, which is responsible for oxidative burst. Rac1, in turn, increases phosphorylation, lessens ubiquitination, and increases the stability of p66Shc protein, consequently resulting in further increase in ROS, thus maintaining cytosolic oxidative stress through mechanisms involving reciprocal regulation through Rac1 (Khanday et al., 2006; Mushtaq et al., 2021). Since the decrease in expression levels of p66Shc protein was already observed, the reciprocal regulation between p66Shc and Rac1 intrigued us to study the effect of quercetin on Rac1 expression. Our results showed that quercetin prevented basal expression of Rac1 and inhibited the expression of transfected Rac1 construct (Figure 3).

Since Rac1 expression is favored by the pro-inflammatory microenvironment (Barth et al., 2009), it could be argued that the decrease in expression of Rac1 could be a consequence of decreased ROS production and *vice versa*. To further look into the mutualistic interdependence between ROS production and Rac1 expression, we monitored the activation of Rac1 following quercetin treatment. Quercetin effectively prevented the activation of Rac1 with a concomitant decrease in ROS production even in transfected Rac1, p66Shc, or Rac1/p66Shc cells (Figures 4, 5). ROS has also been implicated in the metastasis and migration of cancer cells. Although quercetin mediated the prevention of invasion and migration via the downregulation of Rac1 expression and ROS has previously been observed in MCF10A cells (Song et al., 2014), our results gave a direct evidence of quercetin-mediated ROS inhibition in cell migration assays (Figure 6).

**FIGURE 9:**
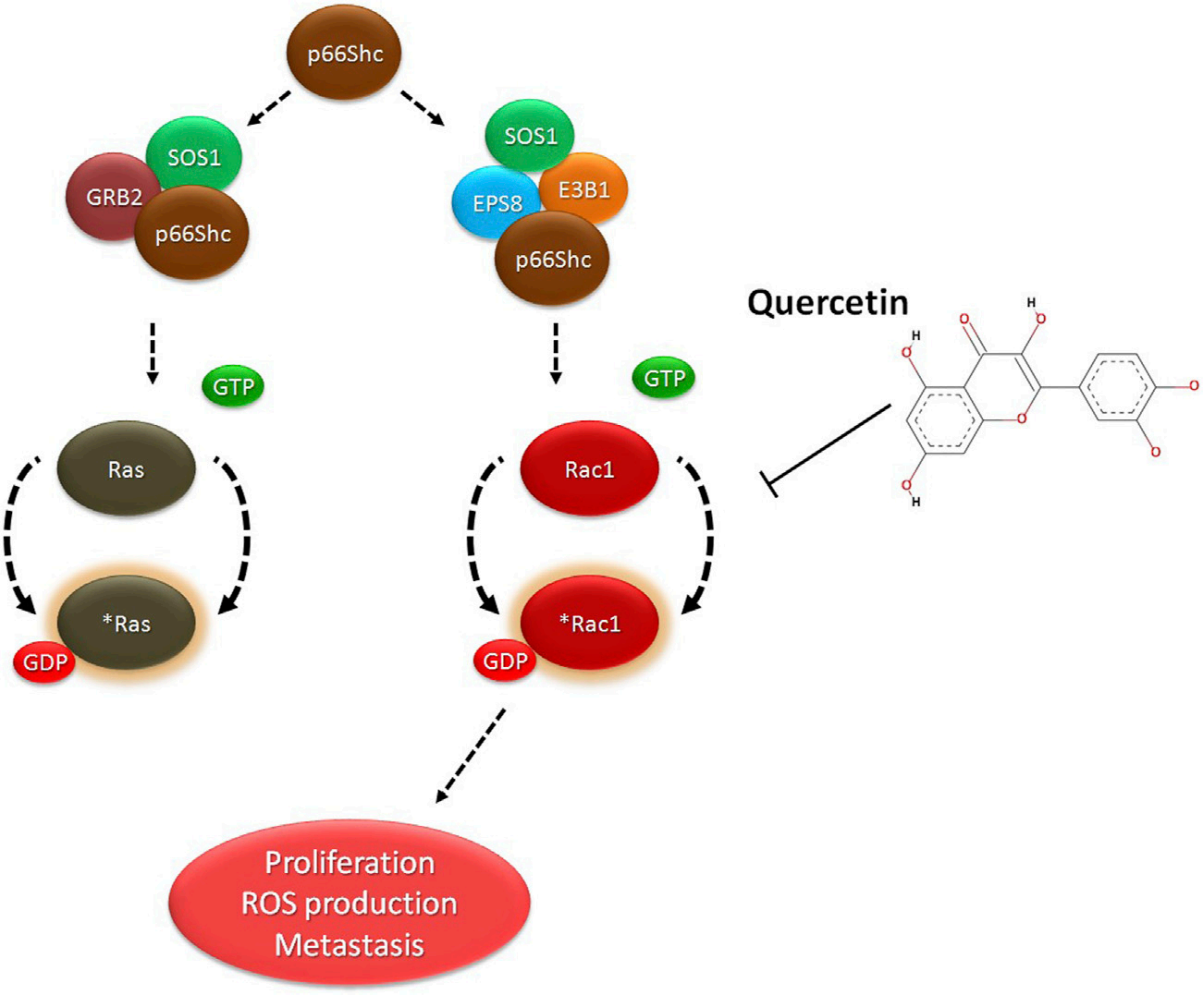
Proposed model of quercetin action: Quercetin binds to Rac1 and decreases cell migration and cell proliferation by inhibiting p66Shc mediated activation of Rac1 and ROS production.

All these findings suggest a targeted inhibition of Rac1 by quercetin, which might possibly prevent Rac1 activation and eventually lead to decreased ROS production and prevent tumor cell migration or metastasis. To investigate such a possibility, computational approaches including molecular docking and thermodynamic-based MD simulation were used. These methods are important tools to understand the functional mechanisms of proteins and other biomolecules in deciphering the structural basis for disease and in the design and optimization of small molecules, peptides, and proteins (Hollingsworth and Dror, 2018; Singh et al., 2022; Bender et al., 2023). We performed molecular docking simulation of Rac1 with quercetin to analyze the mode of inhibition, if any exists. Rac1 is a RhoGTPase and, thus, requires GTP binding for its phosphorylation and subsequent activation. We, therefore, targeted the GTP-binding site of Rac1 with quercetin and also compared it with known Rac1 inhibitors to quantify the extent of possible inhibition by quercetin. In consonance with our postulated theory, quercetin not only prevented the activation of Rac1 by binding efficiently to its GTP-binding site but also depicted the highest extent of inhibition compared to other established inhibitors of Rac1 currently in use (Figure 7; Table 2). MDS studies further confirmed the stability of the Rac1–quercetin complex, validating the high binding energies of the Rac1 and quercetin complex observed in docking studies. The simulation studies further revealed that quercetin binding increases the compactness of Rac1, evident by a decreased radius of gyration and SAS area (Figures 8A–E). Together, *in silico* and *in vitro* findings establish how quercetin modulates Rac1-p66Shc signaling and participates in the control of ROS generation within cells (Figure 9). The study also suggests the modulation of Rac1-p66Shc signaling and scavenging of ROS by quercetin could be prime mechanisms through which dietary flavonoids exert their antioxidant effects, which may play a critical role in their protective action in cancers. In lieu of the achieved results, additional studies are required to understand the role(s) of quercetin on Rac1-p66Shc signaling to establish its role as a therapeutic drug particularly in brain tumors.

**TABLE 2:** Comparative binding interactions of Rac1 with quercetin and the top five inhibitors.

	InSolution™ Rac1 inhibitor	NSC 23766	EHT 1864	6-Mercaptopurine	Ouabain	Quercetin
Binding energy	−4.88	−5.46	−5.22	−5.66	−4.51	−9.86
Ligand efficiency	−0.16	−0.18	−0.15	−0.57	−0.11	−0.45
Inhibition constant (uM)	264.46	99.15	149.4	71.38	490.82	58.82
Intermolecular energy	−8.16	−8.74	−8.5	−5.66	−8.09	−11.65
Hydrogen bonds	0	0	0	2	3	5

Binding energy, degree of ligand interaction/association; ligand efficiency, binding energy per atom of a ligand; inhibition constant, potency of an inhibitor; intermolecular energy, energy of interaction between molecules; hydrogen bonds, intermolecular hydrogen bonds.

## Significance and limitations of the study

Glioma is the most common and aggressive brain tumor having the poorest survival with only 0.05%–4.7% of patients surviving 5 years after diagnosis (Cruz et al., 2022). This tumor is highly invasive and difficult to treat because of blood–brain barrier permeabilization (Cruz et al., 2022). Quercetin not only possesses anticancer activities but can also cross the blood–brain barrier and has potential to treat brain tumors. The present study shows the novel mechanisms exploited by quercetin to target gliomas. We show that quercetin targets the p66Shc/Rac1/ROS pathway, inhibiting tumor growth, and could, thus, be explored in glioma therapeutics. In addition to these findings, there are some limitations associated with this study. One limitation is that this study was carried out only under *in vitro* conditions and needs to be validated under *in vivo* conditions. Another limitation is the less-detailed mechanism explored in this study. The p66Shc/Rac1 pathway has been shown to regulate cell survival, apoptosis, and migration in tumors; therefore, further studies are required before establishing Rac1-p66Shc signaling as a therapeutic target of quercetin.

## Data Availability

The original contributions presented in the study are included in the article/Supplementary Material, further inquiries can be directed to the corresponding authors.
