# Supplementary material for: Quercetin suppresses ROS production and migration by specifically targeting Rac1 activation in gliomas
Source: Front Pharmacol. 2024 Jan 31;15:1318797. doi: 10.3389/fphar.2024.1318797 (PMC10867961; doi:10.3389/fphar.2024.1318797)
Supplement: Supplementary file 1 [file Image1.pdf]

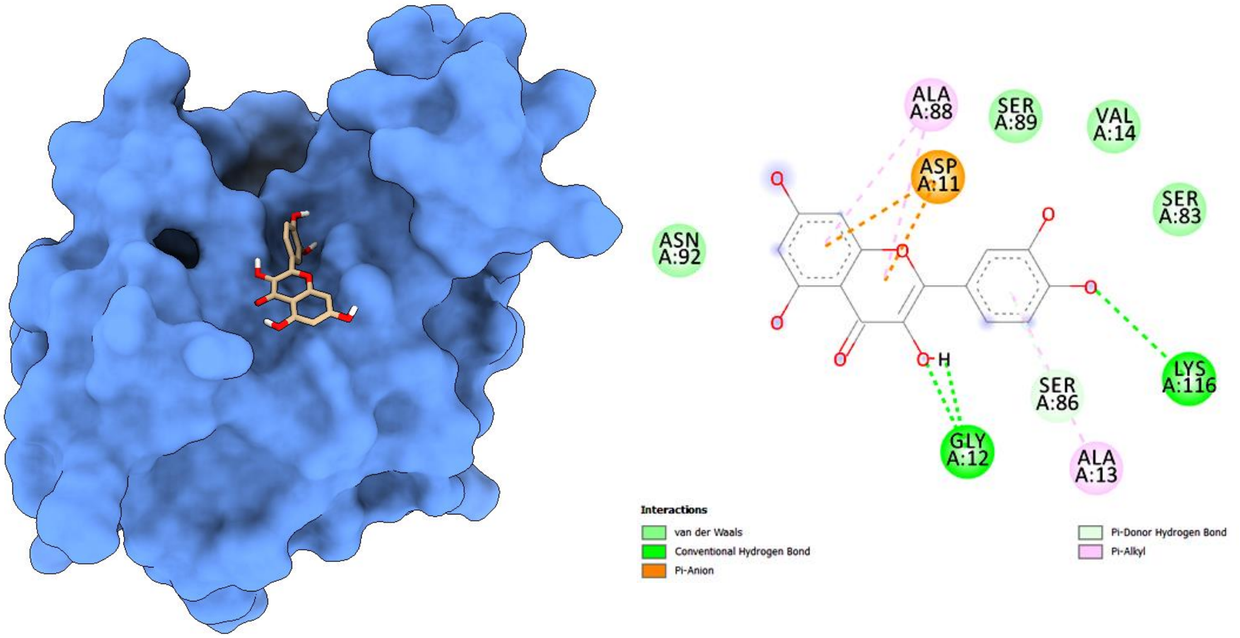

Figure 1. Molecular surface view of the 3TH5 with QUERCETIN bound in deep cavity. 2D interaction is exhibiting the interactions between ligand and protein and dotted lines exhibiting interactions.
